# Quantitative proteomics reveals serum proteome alterations during metastatic disease progression in breast cancer patients

**DOI:** 10.1186/s12014-024-09496-3

**Published:** 2024-07-29

**Authors:** Jaspreet Kaur, Sung Yun Jung, Marie Austdal, Aaditya Krishna Arun, Thomas Helland, Gunnar Mellgren, Tone Hoel Lende, Emiel A. M. Janssen, Håvard Søiland, Ritu Aneja

**Affiliations:** 1 Department of Biology, Georgia State University, Atlanta, GA 30303 USA; 2 Department of Biochemistry and Molecular Pharmacology, Baylor College of Medicine, Houston, TX USA; 3 Department of Pathology, Stavanger University Hospital, Stavanger, Norway; 4 Jan and Dan Duncan Neurological Research Institute, Baylor College of Medicine, Houston, TX USA; 5 Hormone Laboratory, Department of Medical Biochemistry and Pharmacology, Haukeland University Hospital, Bergen, Norway; 6 Department of Clinical Science, University of Bergen, Bergen, Norway; 7 Department of Surgery, Stavanger University Hospital, Stavanger, Norway; 8 Department of Chemistry, Biosciences and Environmental Engineering, University of Stavanger, Stavanger, Norway; 9 Department of Research, Stavanger University Hospital, Stavanger, Norway; 10 Department of Nutrition Sciences, School of Health Professions, University of Alabama at Birmingham, Birmingham, AL 35294 USA

## Abstract

**Background:**

Tumor recurrence and metastatic progression remains the leading cause for breast cancer related mortalities. However, the proteomes of patient- matched primary breast cancer (BC) and metastatic lesions have not yet been identified, due to the lack of clinically annotated longitudinal samples. In this study, we evaluated the global-proteomic landscape of BC patients with and without distant metastasis as well as compared the proteome of distant metastatic disease with its corresponding primary BC, within the same patient.

**Methods:**

We performed mass spectrometry-based proteome profiling of 73 serum samples from 51 BC patients. Among the 51 patients with BC, 29 remained metastasis-free (henceforth called non-progressors), and 22 developed metastases (henceforth called progressors). For the 22 progressors, we obtained two samples: one collected within a year of diagnosis, and the other collected within a year before the diagnosis of metastatic disease. MS data were analyzed using intensity-based absolute quantification and normalized before differential expression analysis. Significantly differentially expressed proteins (DEPs; absolute fold-change ≥ 1.5, *P-*value < 0.05 and 30% abundance per clinical group) were subjected to pathway analyses.

**Results:**

We identified 967 proteins among 73 serum samples from patients with BC. Among these, 39 proteins were altered in serum samples at diagnosis, between progressors and non-progressors. Among these, 4 proteins were further altered when the progressors developed distant metastasis. In addition, within progressors, 20 proteins were altered in serum collected at diagnosis versus at the onset of metastasis. Pathway analysis showed that these proteins encoded pathways that describe metastasis, including epithelial–mesenchymal transition and focal adhesion that are hallmarks of metastatic cascade.

**Conclusions:**

Our results highlight the importance of examining matched samples from distant metastasis with primary BC samples collected at diagnosis to unravel subset of proteins that could be involved in BC progression in serum. This study sets the foundation for additional future investigations that could position these proteins as non-invasive markers for clinically monitoring breast cancer progression in patients.

**Supplementary Information:**

The online version contains supplementary material available at 10.1186/s12014-024-09496-3.

## Introduction

Systemic disease is the cause for BC related mortality [1, 2]. About 6% of patients with BC are diagnosed with metastatic disease, and nearly 30% of women diagnosed with an early-stage BC eventually develop metastasis [1, 3]. Approximately 10–15% of women with metastatic breast cancer die due to metastasis or tumor recurrence [4]. Clinical practice lacks accurate methods to predict the risk of metastasis and effective treatments to prevent metastasis. This is mainly due to paucity in molecular profiles such as proteomics data that compare patient matched metastasis with primary tumor, a challenge resulting from lack of clinically well-annotated and longitudinally derived samples from the patients. The identification of protein markers holds promise for the development of robust prediction tools and effective interventions for patients who have not yet developed detectable metastases and for those with advanced disease.

Currently, there is no clinical guide, or consensus even among the experts, with respect to using these markers for early diagnosis of breast cancer and metastatic progression. The only internationally accepted breast cancer-related biomarker is Carcinoembryonic antigen (CEA) or CA 15 − 3. Thus, there is a pressing need to identify novel prognostic and predictive biomarkers to guide clinical decision-making and improve patient outcomes.

In this study, we evaluated the global-proteomic landscape of BC patients with and without distant metastasis as well as compared the proteome of distant metastatic patients with their corresponding primary tumor. These samples were derived from the Prospective Breast Cancer Biobanking (PBCB) study (NCT04488614) [5]. Our results reveal proteins that are altered at time of diagnosis between patients who progressed to metastasis (progressors) versus those who did not (non-progressors), with a subset of these proteins altered upon establishment of metastatic lesions. The majority of these proteins encode pathways describing cell adhesion, immune suppression, and epithelial mesenchymal transition (EMT) that are hallmarks of metastatic cascade.

## Methods

### Patients and patient samples

The study protocol was approved by all appropriate Institutional Review Boards and was in compliance with material transfer guidelines and data use agreements between Georgia State University and the participating institutes. The study was conducted in accordance with International Ethical Guidelines for Biomedical Research involving human subjects. Written informed consent was obtained from all participants. For MS analysis, serum from 51 breast cancer patients was collected from the Prospective Breast Cancer Biobank (PBCB) project, which enrolled patients at Stavanger University Hospital, Stavanger, Norway and Haukeland University Hospital, Bergen, Norway between 2012 and 2020.

**Fig. 1:**
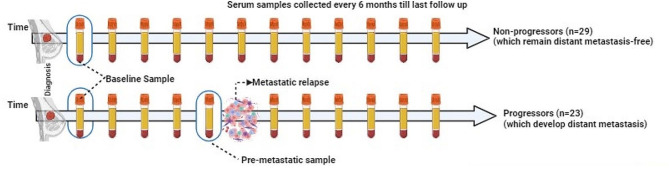
Prospective Breast Cancer Biobanking (PBCB) study: sampling workflow for proteomics analysis. Three distinct samples were examined in the study. These included diagnostic serum samples collected from non-progressors and progressors, and pre-metastatic samples collected from progressors (all indicated in blue boxes)

The study design including patient and patient samples is summarized in Fig. 1. Twenty-nine serum samples were obtained from patients with BC who did not progress to metastasis (also referred to as non-progressors), and 42 samples were obtained from 22 patients who were later diagnosed with distant metastases (progressors). For the non-progressors, one sample was collected within one year after diagnosis was analyzed. For each patient with metastasis, we analyzed two samples: one collected within a year after diagnosis (diagnostic serum sample) and the other collected within one year before the development of metastasis (pre-metastatic serum sample). Pre-metastatic sample was missing for two patients with metastasis, however, these patients had duplicate diagnostic samples.

Patients without distant metastasis were matched to those with distant metastasis according to tumor site, age (± 5 years), chemotherapy, and hormone receptor status. The median age at diagnosis for non-progressors was 55 years, and the median follow-up time was 73.5 months. The median age at diagnosis for progressors was 54 years, and the median follow-up time was 62 months. The clinicopathological characteristics of the patients are summarized in Table 1. Importantly, patients who developed metastasis and those that did not progress further were matched to the extent possible for their grade and presence of lymph node status, at the time of serum collection. All the non-progressors were alive at the time of last clinical follow up. In contrast, about 50% of the progressors were alive with distant metastasis and the rest were dead during the same follow up period.

**Table 1:** Clinicopathological characteristics of breast cancer patients

Baseline Characteristics	Progressors	Non-progressors
**Patient Age, ** ***n*** **(%)**		
30–39	3 (13.6)	2 (6.8)
40–49	3(13.6)	9 (31.0)
50–59	7 (31.8)	8 (27.5)
60–69	5 (22.7)	8 (27.5)
70+	4 (18.1)	2 (6.8)
**Tumor Grade, n (%)**		
I	3 (13.6)	3 (10.3)
II	3 (13.6)	8 (27.5)
III	15 (68.1)	17 (58.6)
Missing	1 (4.5)	1 (3.4)
**Receptor status, n (%)**		
ER/PR/HER2-positive	2 (9.0)	5 (17.2)
HER2-positive	3 (13.6)	3 (10.3)
ER/PR-positive & HER2-negative	6 (27.2)	12 (41.3)
TNBC	6 (27.2)	6 (20.6)
Others	5 (22.7)	3 (10.3)
**LN status, n (%)**		
Positive	14 (63.6)	12 (41.3)
Negative	8 (36.3)	16 (55.1)
Missing	0 (0.0)	1 (3.4)
**Survival Status, n (%)**		
Alive	11 (50.0)	29 (100.0)
Dead	11 (50.0)	0 (0.0)

### MS-based serum proteomics

The mass spectrometer-based proteome profiling of serum was carried out as previously described [6]. . Briefly, the serum was thawed at 37^o^C and 10 µl was incubated with the top 12 abundant serum protein depletion kit (Thermo Scientific Pierce, Cat# 85,164) and digested with trypsin on S-Trap column (ProtiFi, NY). Thereafter, the digested peptides were eluted and vacuum dried. The peptides were then fractionated using the high pH STAGE method [7] into single pools, which was then vacuum dried.

The dried peptide samples were analyzed on an Orbitrap Fusion mass spectrometer (Thermo Fisher Scientific) coupled with an Easy-nLC 1000 nanoflow LC system (Thermo Fisher Scientific). An in-house trap column (2 cm × 100 μm i.d.) and a 5 cm × 150 μm capillary separation column packed with 1.9 μm Reprosil-Pur Basic C18 beads (Dr. Maisch, r119.b9.) were used for nano-HPLC separation in a discontinuous gradient of 4–26% acetonitrile, 0.1% formic acid at a flow rate of 800 nL/min.

The mass spectrometer was operated in a data-dependent mode, acquiring fragmentation spectra of the top 30 strongest ions under the control of Xcalibur software version 4.1 (Thermo Fisher Scientific). The parental ion was acquired in the Orbitrap with a full MS range of 300–1400 m/z at a resolution of 120,000. Higher-energy collisional dissociation (HCD) fragmented MS/MS spectra were acquired in the ion trap with rapid scan mode. The MS/MS spectra were searched against the target-decoy Human RefSeq database (release Jan. 21, 2020, containing 80,872 entries) in Proteome Discoverer 2.1 (Thermo Fisher Scientific) with the Mascot algorithm (Mascot 2.4, Matrix Science). The precursor mass tolerance was 20 ppm, and the fragment mass tolerance was 0.5 Da. Two maximum missed cleavages and dynamic modifications of acetylation of the N-terminus and oxidation of methionine were allowed. Assigned peptides were filtered with a 1% false discovery rate (FDR) using Percolator validation based on q-value.

The MS/MS spectra were searched against the target-decoy Human RefSeq database (release Jan. 21, 2020, containing 80,872 entries) in Proteome Discoverer 2.1 (Thermo Fisher Scientific) with the Mascot algorithm (Mascot 2.4, Matrix Science). The precursor mass tolerance was 20 ppm, and the fragment mass tolerance was 0.5 Da. Two maximum missed cleavages and dynamic modifications of acetylation of the N-terminus and oxidation of methionine were allowed. Assigned peptides were filtered with a 1% false discovery rate (FDR) using Percolator validation based on q-value.

The Peptide Spectrum Matches (PSMs) output from Proteome Discoverer 2.1 was used to group peptides onto the gene level using the ‘gpGrouper’ algorithm (1). An in-house program, gpGrouper, uses a universal peptide grouping logic to accurately allocate and provide MS1-based quantification across multiple gene products. Protein quantification was performed using the label-free, intensity-based absolute quantification (iBAQ) approach and then normalized to FOT (a fraction of the total protein iBAQ amount per experiment). FOT was defined as an individual protein’s iBAQ divided by the total iBAQ of all identified proteins within one experiment.

### Statistical analysis

Missing values in the proteome recovery were replaced with half of the minimally detected value in the entire dataset. After log2 transformation of this dataset, differential analysis (t-test) was performed comparing the proteome of (i) non-progressors and progressors at diagnosis, and (ii) diagnostic and pre-metastatic serum within the progressors. Any protein was deemed to have statistically altered expression if it had a p-value of < 0.05, greater than 1.5 linear fold change, and was detected in over 30% of the samples in any clinical group (differentially expressed proteins or DEPs).

Volcano plots were used to display DEPs (i) at diagnosis by comparing serum samples from patients with BC who did not develop metastasis (non-progressors) vs. patients who eventually developed metastatic progression (progressors) over the period of clinical follow-up and (ii) during progression to metastatic disease by comparing matched serum samples collected at diagnosis with samples collected prior to onset of metastatic disease in the progressors. The latter involved two comparisons: a nested comparison where proteins altered in the diagnostic samples were examined for their expression in the pre-metastatic samples collected from progressors, and a second global comparison that examined for additional proteins that were also altered between the diagnostic and pre-metastatic samples within the progressors. The selected DEPs were analyzed using Advaita Bio’s iPathwayGuide, a systems biology approach for pathway level analysis [8].

### Survival analysis

To assess proteins that may be associated with overall survival, we used Kaplan–Meier method and the difference was tested using the log-rank test on the serum samples collected at diagnosis from progressors who later developed distant metastasis. Patients were divided into two groups based on their protein expression level: median high (expression above median) vs. median low (expression below median). Proteins with missing values greater than 10% were excluded. p-values less than 0.05 were considered as significantly different. Clinical variables analyzed with p-value less than 0.05 using single variant analysis were chosen to enter Cox regression multivariate analysis. The SPSS 22.0 software (IBM Corp.) and the R package “survival” were used for survival statistical tests.

### Data availability

The mass spectrometry data for proteome profiling have been deposited via the MASSIVE repository (MSV000094255) to the Proteome X change Consortium (http://proteomecentral.proteomexchange.org) with the dataset identifier PXD050451).

## Results

### Distinct serum proteomic profile in breast cancer patients

Using an established miniaturized label-free proteome profiling platform, we detected a total of 967 proteins from matched diagnostic and metastatic serum obtained from 22 patients, and diagnostic serum collected from 29 patients who did not exhibit distant metastasis (Table S1). The dynamic range of the proteome data was over seven orders of magnitude (Figure S1 A). Principal component analysis (PCA) of the proteome data revealed that the patients who had localized disease were separated well from those who progressed to metastasis (Figure S1 B). Gene ontology (GO) analysis of 967 detected proteins showed that extracellular regional proteins, including extracellular exosomes and vesicles, were highly enriched in the GO cellular component category (Fig S1 C). Cell adhesion, response to stress, wound healing, response to wound healing, and coagulation were also highly enriched in the GO biological process category (Figure S1 D).

**Fig. 2:**
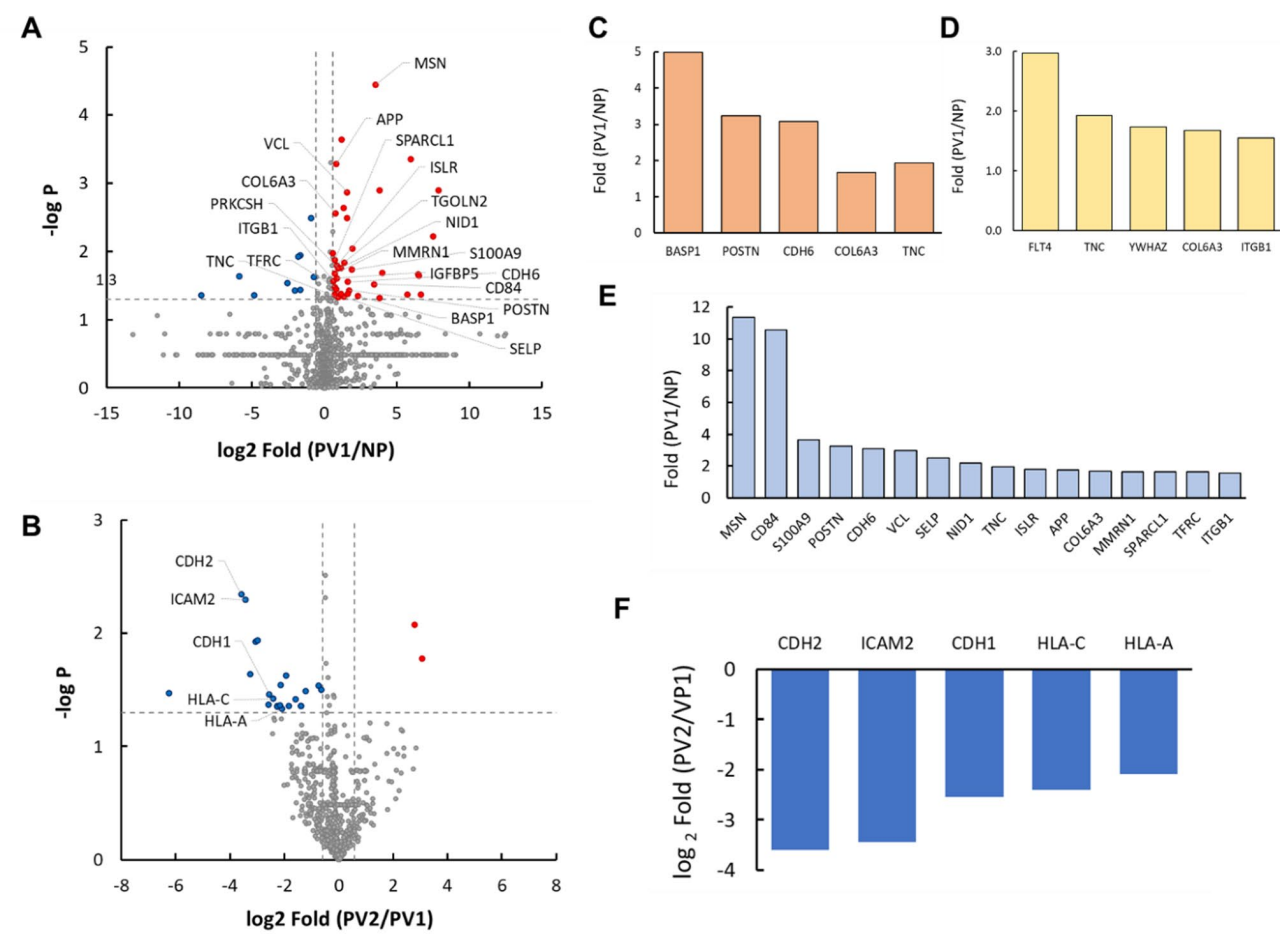
Distinct serum proteomic profile in breast cancer patients. (**A**) Volcano plot showing significantly altered proteins (see text) in serum of progressors at diagnosis (PV1) vs. non-progressors (NP), (**B**) same as in **A**, but comparing serum collected at diagnosis (PV1) vs. prior to metastasis within progressors (PV2), (**C**) Bar plot showing proteins that are enriched for epithelial mesenchymal transition (EMT) comparing PV1 vs. NP, (**D**) same as in **C**, but for proteins that are enriched for KEGG pathways describing focal adhesion induced PI3K-Akt signaling, (**E**) same as in **C**, but for proteins that are enriched for cell adhesion concept comparing PV1 vs. NP, (**F**) bar plot showing proteins that were involved in decreased enrichment of cell adhesion proteins comparing serum from PV1 vs. PV2

We further looked for differential proteins that distinguished progressors (PV1) from non-progressors (NP) at diagnosis. Differential proteins were selected if they had a *p* < 0.05, fold change > 1.5, and minimum 30% concurrence in any of the two groups being compared. A total of 32 proteins were statistically significantly increased, while 7 proteins were significantly decreased in progressors compared to non-progressor at diagnosis (Fig. 2A, Table S3). Additionally, a similar analysis comparing patient matched serum collected at diagnosis (PV1) and upon onset of metastasis (PV2) revealed that 2 proteins were significantly increased, and 18 proteins were significantly decreased (Fig. 2B, Table S4). The biological and physiological importance of these significantly altered proteins was further analyzed using iPathwayguide™ program (Fig. 2C-F).

As shown in Fig. 2C, we found that differentially expressed proteins comparing progressors to non-progressors at diagnosis consisted of a subset that enriched for the epithelial-mesenchymal transition concept (EMT, GSEA M5930). The proteins in this subset included BASP1, POSTN, CDH6, COL6A3, and TNC. In addition, an included subset of downregulated proteins comparing progressors to non-progressors at diagnosis enriched for the cell adhesion concept (GO: 000715). These proteins included MSN, CD84, S100A9, POSTN, CDH6, VCL, SELP, NID1, TNC, ISLR, APP, COL6A3, MMRN1, SPARCL1, TFRC, and ITGB1 (Fig. 2E).

Furthermore, KEGG pathway analysis results of proteins elevated in the diagnostic serum of progressors vs. non-progressors namely FLT4, TNC, YWHAZ, COL6A3, and ITGB1, can suggest activation of the focal adhesion-induced PI3K-Akt signaling pathway (KEGG:04151) (Fig. 2D). Importantly, proteins involved in cell adhesion (KEGG:04514) namely CDH2, ICAM2, CDH1, HLA-C, and HLA-A (Fig. 2F) were downregulated in serum samples collected at metastasis (PV2) compared to patient matched samples collected at diagnosis (PV1).

To confirm the detection of differentially regulated proteins identified in the discovery phase of our proteomics study, we conducted targeted Parallel Reaction Monitoring (PRM) mass spectrometry. We selected one to three peptides from proteins that were found to be more abundant in PV1 samples compared to non-metastatic patient (NP) samples, as illustrated in Figure S1. The PRM analysis results confirmed the findings obtained from the discovery runs.

**Fig. 3:**
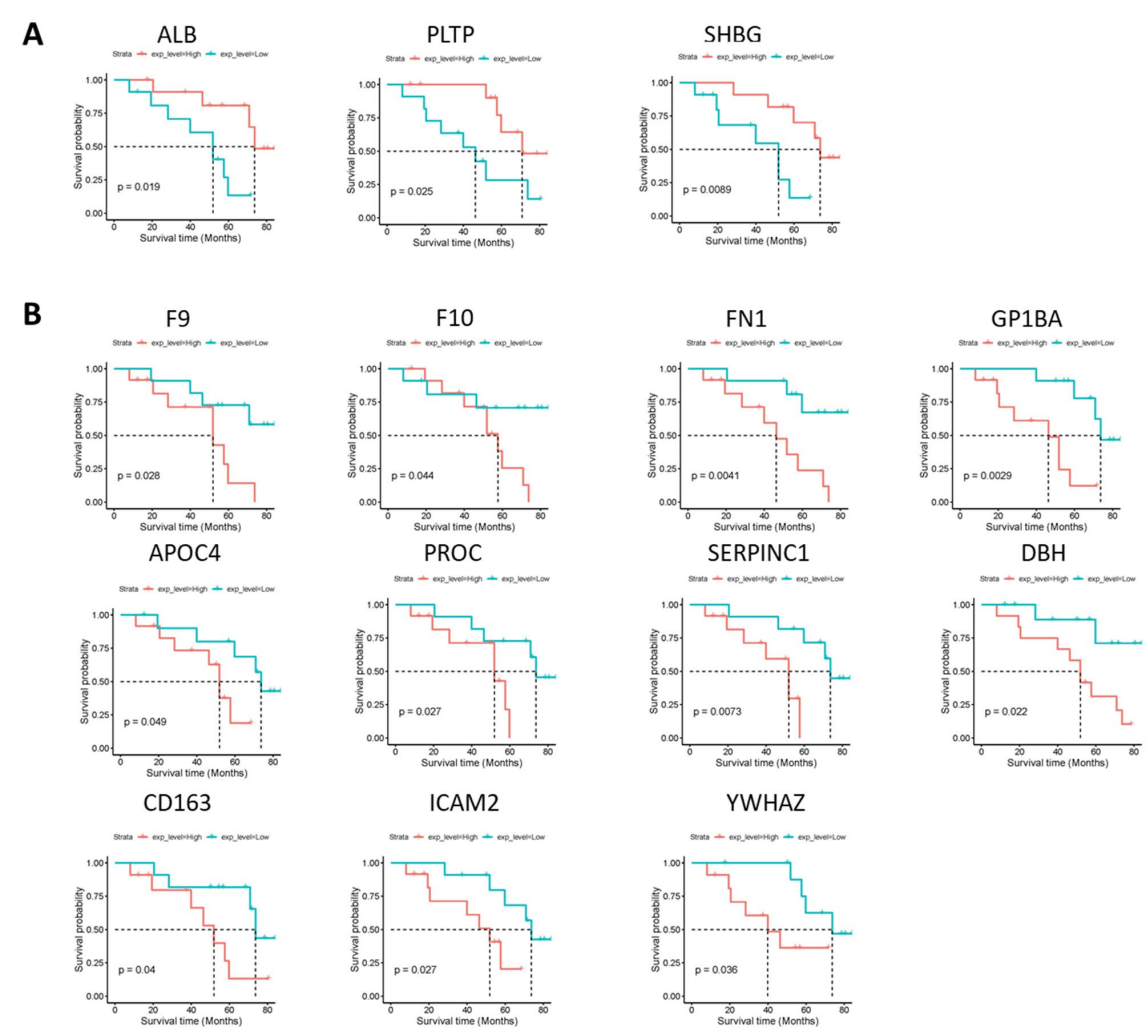
Kaplan-Meier plots (KM plot) describing the prognostic value of differentially expressed proteins comparing PV1 (progressors) and NP (non-progressors) collected at diagnosis. (**A**) KM plot of proteins whose increased expression in the diagnostic serum was significantly associated with poor clinical outcome. (**B**) same as in (**A**), but for proteins whose decreased expression in diagnostic serum was significantly associated with poor clinical outcome

We then assessed proteins that could possibly be associated with overall survival in patients with metastasis using the Kaplan-Meier (log-rank) test. As shown in Fig. 3, we found elevated expression of ALB, PLTP, and SHBG to be positively correlated with overall survival of the patients (Fig. 3A). Along similar lines, expression of CD163, F9, F10, GP1B, ICAM2, APOC4, PROC, SERPINC1, DBH, and YWHAZ, were negatively correlated with overall survival of patients (Fig. 3B).

## Discussion

We present a comparison of the proteomic landscape in sera from breast cancer patients who did not progress to metastasis (non-progressors) and who eventually developed metastasis (progressors) during the period of clinical follow up. Serum samples were collected from progressors once at diagnosis, and once prior to onset of metastatic disease. The latter forms the highlight of the study where protein profiles associated with disease progression have been identified in a clinical trial cohort by comparing matched serum from diagnostic vs. metastatic setting within the same patient, thus mitigating patient heterogeneity [5]. In light of this, we believe that our study has the strength to advance diagnostic and preventative clinical practices for breast cancer treatment by providing important data for the discovery of blood-based biomarkers associated with metastatic progression.

The analysis of significantly altered proteins comparing non-progressors and progressors at diagnosis shows a very clear increase in the proteins which are involved in EMT, focal adhesion, and cell adhesion. Epithelial mesenchymal transition (EMT) is tightly involved in cancer progression and a key step in the metastatic cascade [9]. Cell adhesion proteins serve as pivotal proteins residing on the cell’s surface, facilitating the binding of cells to both their counterparts and the extracellular matrix (ECM). ECM proteins are implicated in mediating the adhesion of circulating tumor cells to the endothelium of distant organs, a crucial step in metastasis.

Numerous studies have indicated that the regulation of protein expression for markers of EMT and cell adhesion can be perceived as drivers of tumor progression. For example, a study by Ünlü et al. [10] demonstrated that the inhibition of tissue factor (TF) signaling led to a tenfold reduction in metastasis, irrespective of the growth of the primary tumor. TF is the transmembrane heterodimer which serves as adhesion molecules for interactions between cells and extracellular matrix (ECM). This blockade of TF signaling resulted in decreased levels of epithelial-to-mesenchymal transition, reduced cancer stemness, and a decrease in the expression of pro-metastatic markers, namely Slug and SOX9, across multiple breast cancer cell lines [10]. Thus, the detection of these cancer– related bio markers in the diagnostic serum samples early on can be crucial in predicting the likelihood of development of metastasis in patients.

Apart from up-regulation in the levels of EMT and ECM proteins, our data show that the diagnostic serum sample from progressors has higher levels of proteins involved in the focal adhesion-PI3K signaling pathway. Activation of focal adhesion–mediated PI3K/AKT signaling in the cancer metastasis signaling pathway has been demonstrated to be a critical regulator of epithelial-mesenchymal transition (EMT) in colorectal tumor cells in numerous previous publications [11–15]. Our results are consistent with these previous findings.

In this study, we also identified several proteins that showed positive or negative correlations with the overall survival of breast cancer patients. The three proteins that showed a positive correlation between protein levels and overall survival included albumin (ALB), phospholipid transfer protein (PLTP), and sex hormone-binding globulin (SHBG). Among these, the prognostic value of ALB is well-aligned with previous reports. Several studies have shown that low levels of ALB are associated with poor survival in patients with colorectal and breast cancer [16, 17]. This is consistent with our findings that higher levels of ALB can prolong survival in metastatic breast cancer patients. Moreover, there is some evidence that an increased S-SHBG binds to steroid sensitive calls, like breast cancer cells, and inhibits proliferation by activating cAMP regulated down-stream intracellular pathways [18]. However, there is no definitive evidence on the correlation of PLTP with cancer patient survival in previous publications. Although elevated levels of SHBG can serve as early detection markers for gastric cancer, however, this is not the case for breast cancer [19, 20].

Among the proteins that showed a negative correlation with overall survival, fibronectin 1 (FN1) indicates poor prognosis in gastric cancer [21]. Similarly, overall survival and cancer-specific survival in stage I NSCLC is reported to be negatively correlated with YWHAZ [22], which is in line with our finding. In contrast, our data on association of the two glycoproteins (GPs), ICAM2 and SERPINC1 with patient outcome differs from literature reports. Previous studies have shown that downregulation of ICAM2 was correlated with poor prognosis in certain cancers (breast, lung, bladder, and soft tissue cancers) [23], and SERPINC1 expression was positively correlated with overall survival, progression-free survival, relapse-free survival, and disease-free survival of HCC patients [24]. This discrepancy may be due to heterogeneity within the patient population analyzed in the prior reports. Nevertheless, this needs to be verified in future studies.

Among the limitations of this study, first, this dataset is small in terms of diversity of patient samples. Though, the dataset represents samples collected from different centers across the country. Secondly, the analysis is based on one (for non-progressors) and two (for progressors) longitudinal data points, which does not take into account the fluctuations in serum proteins within individuals. A more rigorous study incorporating multiple data points is worth studying in the future. Finally, although the potential biomarkers associated with BC progression were differentially expressed in plasma of BC patients, we could not completely exclude the possibility that other organs or tissues could have contributed to these proteins. Which might need to be explored through comparison among tissues also. A more robust multi-tissue comparison study is warranted in the future.

## Conclusion

In conclusion, we found that proteins involved in EMT, cell adhesion, and the PI3K-AKT pathway are elevated early on in serum of breast cancer patients who eventually progress to metastasis. In light of this, collecting clinically defined samples from unique set of patients matched longitudinally from a clinical trial, we have identified several proteins that are elevated in progressors and associated with poorer clinical outcome, and hence have the potential to be considered as markers of erupting systemic breast cancer disease. Although all of these findings need to be validated using an independent cohort, we strongly believe our findings lays the foundation for additional validation studies could lead to development of a joint network of novel prognostic and predictive biomarkers in serum to predict the likelihood of disease progression at an earlier point in time that we know of today.

### Electronic supplementary material

Below is the link to the electronic supplementary material.

Supplementary Material 1

## Data Availability

No datasets were generated or analysed during the current study.
