# Supplementary material for: Quantitative proteomics reveals serum proteome alterations during metastatic disease progression in breast cancer patients
Source: Clin Proteomics. 2024 Jul 29;21:52. doi: 10.1186/s12014-024-09496-3 (PMC11285292; doi:10.1186/s12014-024-09496-3)
Supplement: Supplementary file 1 — Supplementary Material 1 [file 12014_2024_9496_MOESM1_ESM.docx]

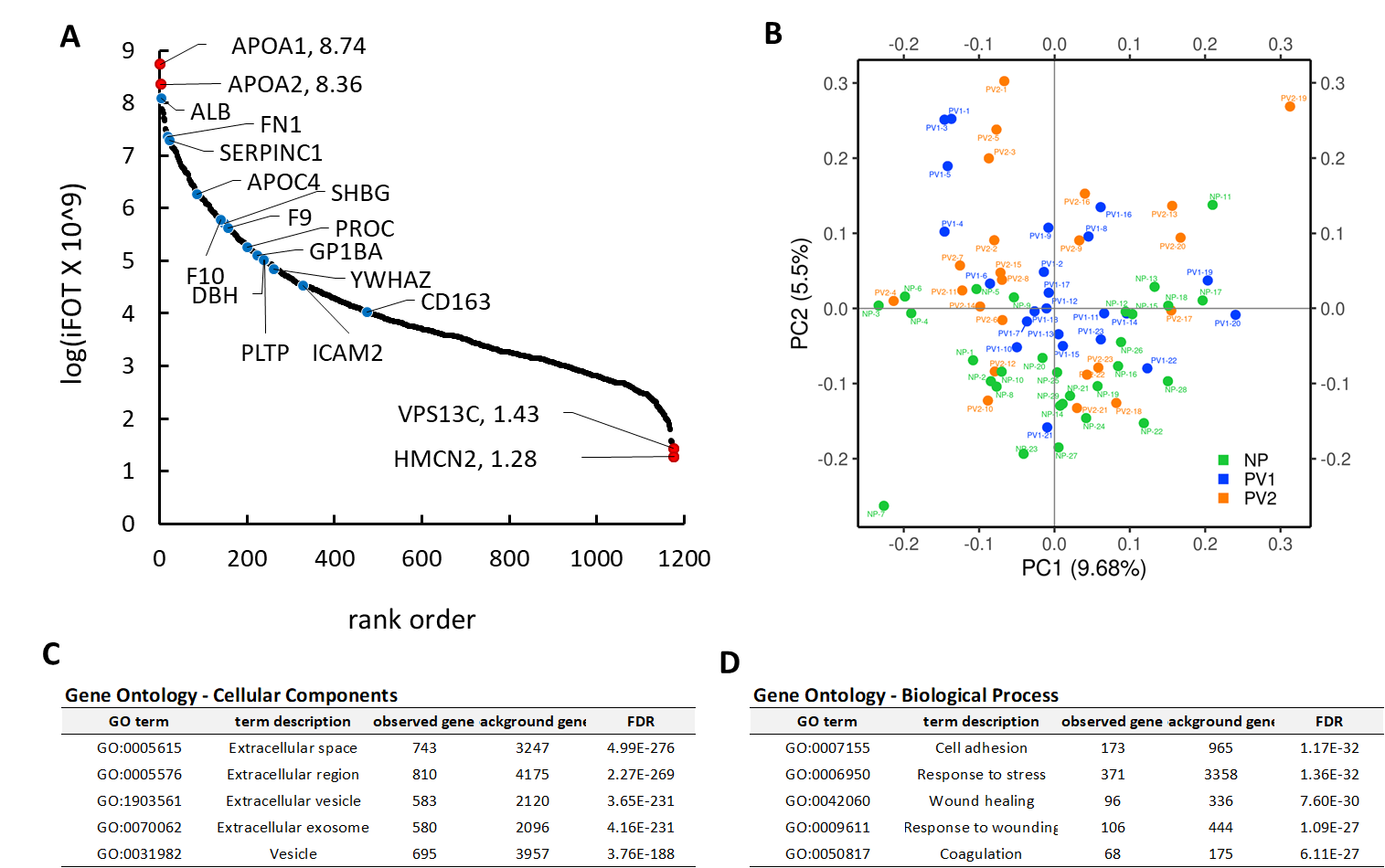


**Figure S1.** A) The dynamic range of the proteome data was over seven orders of magnitude. B) Principal component analysis (PCA) of the proteome data revealed that the non-progressor (NP) samples were distinct from the PV1 and PV2 samples obtained from progressors at diagnosis and upon onset of metastasis, respectively. In contrast, the proteomic profiles were unable to separate PV1 and PV2 samples from each other. Gene ontology (GO) analysis of the 967 detected proteins showed that extracellular regional proteins, including extracellular exosomes and vesicles, were highly enriched in the GO cellular component category. D) Cell adhesion, response to stress, wound healing, response to wounding, and coagulation were also highly enriched in the GO biological process category, as expected for regular serum components.

**
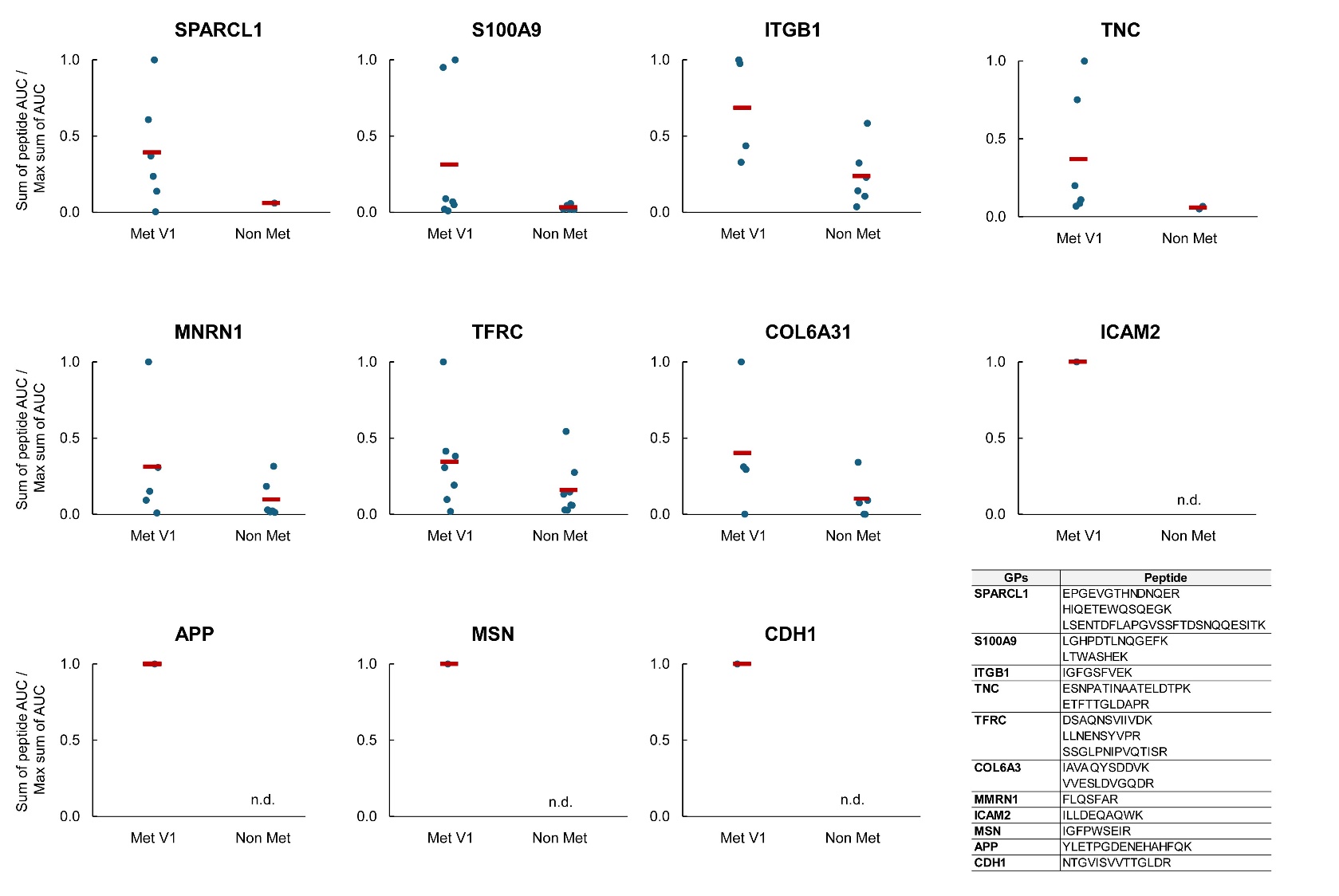
**

**Figure S2.** The PRM result of selected proteins. Protein abundance is represented by the total area-under-curve (AUC) of the tested peptides for each protein. The table inset shows the list of tested proteins and their corresponding peptides. The dots represent the sum of peptide intensities (AUC) per sample divided by the maximum sum of AUC. The red line represents the average protein abundance in each group.

Table S3: The levels of the serum proteins that are Up-or downregulated in progressors compared to non-progressors at diagnosis

| **Gene symbol** | **Gene ID** | **Average paired Log2Fold (Progressor V1/ non-progressor) change** | **Paired P value** |
| --- | --- | --- | --- |
| MSN | 4478 | 3.504 | 0.000 |
| SBSN | 374897 | 1.173 | 0.000 |
| ZFP14 | 57677 | 5.923 | 0.000 |
| APP | 351 | 0.810 | 0.001 |
| PSMA7 | 5688 | 3.818 | 0.001 |
| TKT | 7086 | 7.880 | 0.001 |
| VCL | 7414 | 1.570 | 0.001 |
| ANG | 283 | 1.319 | 0.002 |
| COL6A3 | 1293 | 0.743 | 0.003 |
| FLT4 | 2324 | 1.571 | 0.003 |
| FAH | 2184 | 7.499 | 0.006 |
| DKK3 | 27122 | 1.927 | 0.009 |
| SPARCL1 | 8404 | 0.704 | 0.013 |
| TGOLN2 | 10618 | 1.348 | 0.015 |
| ISLR | 3671 | 0.847 | 0.016 |
| NID1 | 4811 | 1.123 | 0.018 |
| PRKCSH | 5589 | 0.964 | 0.018 |
| S100A9 | 6280 | 1.869 | 0.019 |
| FAM3C | 10447 | 3.980 | 0.021 |
| MMRN1 | 22915 | 0.708 | 0.021 |
| IGFBP5 | 3488 | 0.876 | 0.025 |
| ITGB1 | 3688 | 0.632 | 0.027 |
| CDH6 | 1004 | 1.621 | 0.028 |
| TFRC | 7037 | 0.702 | 0.033 |
| YWHAZ | 7534 | 0.793 | 0.036 |
| POSTN | 10631 | 1.696 | 0.037 |
| PRAP1 | 118471 | 1.608 | 0.042 |
| APMAP | 57136 | 1.144 | 0.042 |
| RARRES2 | 5919 | 0.725 | 0.043 |
| BASP1 | 10409 | 2.317 | 0.045 |
| SELP | 6403 | 1.311 | 0.046 |
| TNC | 3371 | 0.950 | 0.047 |
| C1QB | 713 | -0.903 | 0.003 |
| KRT77 | 374454 | -1.652 | 0.011 |
| KRT6C | 286887 | -1.818 | 0.012 |
| COLEC10 | 10584 | -0.713 | 0.023 |
| KRT72 | 140807 | -2.534 | 0.029 |
| BLVRB | 645 | -1.653 | 0.036 |
| LOC102723407 | 102723407 | -2.036 | 0.037 |

Table S4: The levels of the serum proteins that are Up-or downregulated in matched serum samples collected from the same patient at diagnosis (PV1) and upon onset of metastasis (PV2)

| **Gene symbol** | **Gene ID** | **Average paired Log2Fold (Progressor V2 / Progressor V1) change** | **Paired P value** |
| --- | --- | --- | --- |
| IGFBP2 | 3485 | 3.073 | 0.017 |
| PLA2G7 | 7941 | 2.784 | 0.008 |
| CDH2 | 1000 | -3.594 | 0.005 |
| PCSK9 | 255738 | -2.274 | 0.044 |
| ISLR | 3671 | -2.983 | 0.012 |
| SOD2 | 6648 | -3.251 | 0.023 |
| ACE | 1636 | -2.152 | 0.043 |
| HLA-A | 3105 | -2.090 | 0.046 |
| HLA-C | 3107 | -2.401 | 0.038 |
| SAA4 | 6291 | -6.246 | 0.034 |
| VCL | 7414 | -1.939 | 0.024 |
| YWHAZ | 7534 | -2.255 | 0.045 |
| CDH1 | 999 | -2.551 | 0.035 |
| LYVE1 | 10894 | -2.143 | 0.028 |
| DBH | 1621 | -0.747 | 0.029 |
| ICAM2 | 3384 | -3.439 | 0.005 |
| MST1 | 4485 | -1.210 | 0.032 |
| RARRES2 | 5919 | -3.059 | 0.012 |
| SPARC | 6678 | -0.634 | 0.031 |
| CD44 | 960 | -1.839 | 0.044 |
